# Cross-country collaboration for physical activity promotion: experiences from the European Union Physical Activity Focal Points Network

**DOI:** 10.1093/eurpub/ckac079

**Published:** 2022-08-26

**Authors:** Antonina Tcymbal, Peter Gelius, Karim Abu-Omar, Sven Messing, Stephen Whiting, Kremlin Wickramasinghe

**Affiliations:** Department of Sport Science and Sport, Friedrich-Alexander-Universität Erlangen-Nürnberg, Erlangen, Germany; Department of Sport Science and Sport, Friedrich-Alexander-Universität Erlangen-Nürnberg, Erlangen, Germany; Department of Sport Science and Sport, Friedrich-Alexander-Universität Erlangen-Nürnberg, Erlangen, Germany; Department of Sport Science and Sport, Friedrich-Alexander-Universität Erlangen-Nürnberg, Erlangen, Germany; WHO European Office for the Prevention and Control of Noncommunicable Diseases, Moscow, Russian Federation; EPIUnit—Instituto de Saúde Pública, Universidade do Porto, Porto, Portugal; WHO European Office for the Prevention and Control of Noncommunicable Diseases, Moscow, Russian Federation

## Abstract

**Background:**

An analysis of currently existing partnerships and cross-country collaboration for physical activity (PA) promotion is valuable for understanding how such partnerships operate, and how they impact national PA promotion efforts. This study aimed to outline the formation and development of the European Union’s (EU) Physical Activity Focal Points Network, to evaluate its outputs and benefits and to describe its potential and challenges.

**Methods:**

A mixed methods approach were employed, including document analysis, semi-structured interviews with key officials and an online evaluation survey with the focal points.

**Results:**

The network was founded in 2014. Its main task is to coordinate the national collection of information for the EU’s Health-Enhancing Physical Activity (HEPA) Monitoring Framework. Besides collecting data, focal points usually meet twice a year to share best practices and plan activities for the promotion of PA within the EU. The results of the evaluation survey show that participation in the network helped members specify goals for PA promotion, gain more knowledge regarding how to promote PA, identify more opportunities to promote PA in their country and to join a collaborative project with other countries.

**Conclusions:**

The study shows that the EU Physical Activity Focal Points Network may serve as an example of successful cross-country collaboration in PA promotion. The network has been able to make a contribution to monitoring the implementation of the EU Council Recommendation on HEPA across sectors in particular and of PA promotion in the EU in general.

## Introduction

There is ample scientific evidence that physical activity (PA) is one of the most important components of successful health promotion and disease prevention interventions for individuals and communities.^1^^,^^2^ However, according to the latest estimates, 36.2% adults in the European Union (EU) are physically inactive.^3^ Since it has been shown that certain policies (such as school-based and infrastructural policies) can be effective to increase PA,^4–6^ a number of resources have been developed to support efforts to counter insufficient PA levels, such as the EU PA Guidelines and the PA Strategy for the WHO European Region 2016–2025.^7^^,^^8^ Another example is the WHO Global Action Plan on Physical Activity 2018–2030, which calls for a 10% relative reduction in the prevalence of insufficient PA.^1^ These documents stipulate the use of multiple policy instruments, such as recommendations, regulation and financing mechanisms.^9^ One important instrument mentioned throughout these policy documents is coordination and networking between stakeholders.^1^^,^^8^^,^^10^ This includes the coordination of policymaking among government sectors (e.g. health, sports, education, transport, urban planning, environment and social affairs) and levels (regional, national and local), as well as international cooperation on global health and health challenges of a cross-border nature.

In light of this, an analysis of currently existing partnerships and cross-country collaborations for PA promotion is valuable for understanding how such partnerships operate and how they can support the adoption of policy instruments that would impact national PA promotion efforts. An interesting case example of such a partnership is the Physical Activity Focal Points Network (Focal Points Network) of the EU: In accordance with the EU Physical Activity Guidelines,^7^ the Council of the EU’s ‘Recommendation on Promoting Health-Enhancing Physical Activity across Sectors’ (Council Recommendation on HEPA) outlines 23 indicators (the HEPA Monitoring Framework) to establish and extend monitoring and surveillance of HEPA promotion policy in the 27 Member States.^11^ To support these activities, EU countries were recommended to appoint national HEPA focal points to collect and validate national data. All Member States followed this recommendation, and since 2014, these focal points have usually met twice a year to discuss issues related to the HEPA Monitoring Framework, but also to share best practices and plan activities for the promotion of PA across the EU.

This article will describe the formation and development of the Focal Points Network, present the results of an evaluation survey on the network’s outputs and usefulness, conducted among the network’s members, and discuss its potential and challenges. This will allow deeper insights into what drives governments to set up and participate in such networks, how they can practically be run, and how effective they are.

## Methods

To obtain information about the Focal Points Network, we employed a mixed methods approach with three components:

1. *Document content analysis* of 12 network meeting reports (2014–2020) prepared and provided by the WHO Regional Office for Europe. The analysis included collecting information on meeting agendas and the composition of participants.
2. *Semi-structured interviews* with the head of the WHO European Office for the Prevention and Control of Noncommunicable Diseases at the WHO Regional Office for Europe (conducted on 18 September 2020) and the responsible policy officer in the Sport Unit of the European Commission’s (EC) Directorate-General for Education and Culture (conducted on 12 October 2020). Both interviews included questions about the network’s origins, establishment and development, the evaluation of benefits and outputs and potential directions of future development.
3. *An online evaluation survey* conducted among the national PA focal points. WHO Regional Office for Europe, together with the WHO Collaborating Centre for Physical Activity and Public Health at FAU Erlangen-Nürnberg (Germany), developed an online questionnaire on the effectiveness of and satisfaction with the Focal Points Network. The survey was designed to reflect focal points’ experiences with the network as well as its impact on HEPA promotion in their countries. An invitation was sent via email to 39 focal points from all Member States by the WHO Regional Office for Europe in May 2020. Focal points had 2 weeks to provide answers, and an additional reminder to participate in the survey was sent 5 days before the deadline.

The document analysis was used to identify network structures and to track meetings. Interview data and document analysis were used to trace the origins of the network and to document its establishment. The online survey was used to assess participants’ opinion about the outputs and benefits of the network.

## Results

### Network structure

In 2013, EU Member States adopted the Council Recommendation on HEPA,^11^ which was based on the EU Physical Activity Guidelines^7^ issued in 2008 and aimed to encourage Member States to implement cross-sectoral national PA policies, integrating policy areas such as sport, health, education, environment and transport. The Council Recommendation includes a set of 23 HEPA indicators to monitor the current state and progress of HEPA promotion and policies. EU Member States were requested to appoint national PA focal points within 6 months to support the monitoring of HEPA indicators at the national level. Similar networks are also found in other EU policy areas, for instance nutrition (Network of European Food Safety Authority Focal Points)^12^ or occupational health (European Agency for Safety and Health at Work).^13^ As described in the Council Recommandation, a HEPA focal point is ‘the main contact person in a Member State for providing information and data corresponding to the indicators’. In addition, focal points should ‘facilitate interdepartmental cooperation on HEPA policies’ and should ‘cooperate closely among themselves and with the Commission by engaging in a process of regular exchange of information and best practices on HEPA promotion in the relevant Union level structures for sport and for health as a basis for strengthened policy coordination’. Member states are requested to ‘inform the Commission of their [i.e. the focal points’] appointement’.

In order to initiate an exchange between these focal points, the EC Directorate-General for Education and Culture (DG EAC), with support from the WHO Regional Office for Europe’s Division of Noncommunicable Diseases and Promoting Health through the Life-course, founded the EU Physical Activity Focal Points Network in 2014. In general, the EC has taken on a more organizational role, while WHO was more responsible for scientific support. The participation of WHO was funded by a direct grant from the EU to WHO to provide technical support for the promotion of PA and sports. The overall composition of the Focal Points Network and its development over time are described in table 1.

**Table 1:** List of national representatives on the Focal Point Network meetings

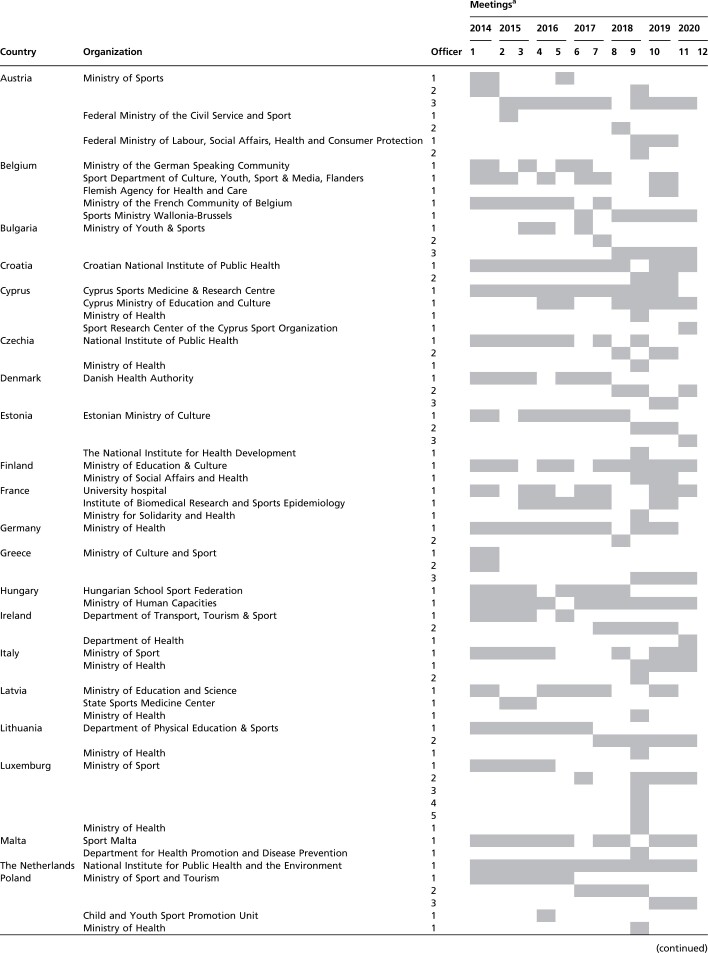 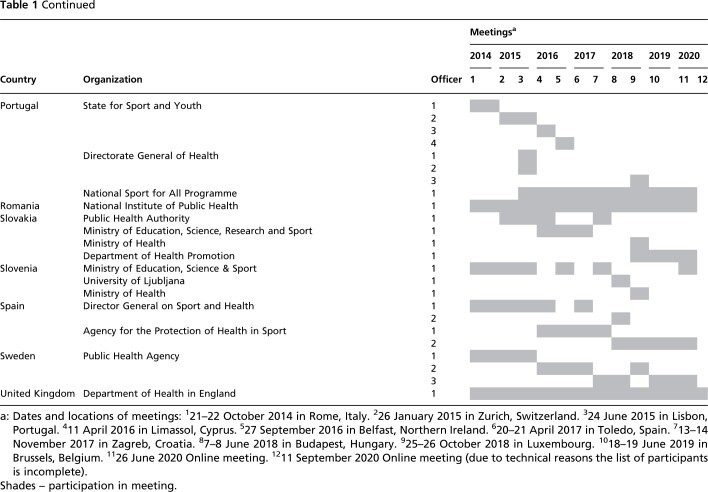

### Focal points network meetings

To facilitate the sharing of experiences and discussions about PA policy monitoring, new projects and collaborative activities, regular meetings of the Focal Points Network have been held since 2014, jointly organized by the EC (DG EAC, Sport Unit) and the WHO Regional Office for Europe. In total, ten personal meetings took place between October 2014 and June 2019 in ten different countries of the European region. In 2020, due to the COVID-19 pandemic, two meetings were held online.

### Participation in focal points network meetings

While participation in the Focal Points Network meetings in not mandatory, each EU Member State usually nominates at least one representative to participate in these meetings. Some Member States are represented by more than one focal point, for instance from different regions (e.g. Belgium has three focal points from Flanders, Wallonia and the German speaking community) or sectors (e.g. Cyprus is represented by focal points from the Sports Medicine and Research Centre as well as the Ministry of Education and Culture). In three cases (the Netherlands, Romania, and the UK), the same individual acted as Member State representative for all the meetings. Two individuals took turns acting as focal points in five countries (Croatia, Finland, Germany, Hungary and Malta). In the 20 remaining cases, a team of three or more individuals took turns acting as focal points. The majority of Member States (*n* = 15) tended to nominate individuals from the same organization. Member States were most often represented at the meetings by officials from health sector (11 Ministries of Health and 18 other organizations related to health sector). Ten Member States were at least once represented by their Ministry of Sport, and deputies of 12 other organizations from the sports sector were engaged in the network. In addition, representatives of nine other ministries participated in the meetings. In some cases, the focal point changed at some point along the way due to a change of government or ministry portfolios. If they cannot attend meetings themselves, focal points will usually nominate a colleague from their own institution as a deputy. However, five Member States missed one meeting, and nine other Member States missed more than one meeting (excluding the last meeting with incomplete list of participants).

### Topics of the focal points network meetings

The main discussion topics were obtained from the official meetings minutes (see table 2, topics similar in content were combined). The most common topics were the monitoring of the 23 HEPA indicators, the resulting PA Country Factsheets^14–16^ as well as general aspects of PA surveillance, PA recommendations and PA promotion for children. Meeting agendas usually also included presentations on ongoing projects at the EU/WHO level and the exchange of national good practices.

**Table 2:** Main topics of the Network meetings

	Meetings											
	2014	2015		2016		2017		2018		2019	2020	
Topics	1	2	3	4	5	6	7	8	9	10	11	12
**Policy context and background documents**												
EU policy context for HEPA	X				X							
The WHO strategy on PA for health for European Region	X		X	X								
Policy development for PA promotion	X											
New WHO Global Action Plan for PA								X				
**Surveillance, Monitoring and Good practice**												
HEPA Monitoring Framework (23 HEPA Indicators)	X	X	X	X		X	X	X				
Areas of support for Member States		X	X									
The epidemiology of physical inactivity/surveillance	X	X			X	X	X	X				
WHO European database on nutrition, obesity and PA	X	X	X	X								
Good practice interventions to promote PA	X								X			
Presentations on ongoing projects at the EC/WHO level				X	X	X	X			X		
HEPA PAT—a PA policy audit tool			X									
PA Country Factsheets			X	X			X	X	X			
**PA recommendations**												
PA recommendations			X	X						X		
PA recommendations for children under 5										X		
**Specific topics and population groups**												
Addressing equity issues in PA for health policy	X											
PA in the school/children					X	X	X	X	X			X
PA in elderly populations						X				X		
Urban planning and active mobility							X			X		
The impact of the COVID-19 pandemic on health and PA promotion in the EU											X	
The effects of COVID-19 on PA levels of children, currently PE regulations												X

X – meetings where this topic was discussed.

### Network outputs

According to the Council Recommendation on HEPA,^11^ the main task of the Focal Points Network is to coordinate the national collection of information for the HEPA Monitoring Framework every 3 years. Consequently, surveys among the focal points to collect these data were jointly conducted by the EC and WHO in 2015, 2018 and 2021. Based on these surveys, the WHO Regional Office for Europe published PA Country Factsheets for the EU Member States for all three waves.^14–16^ These publications provide a general overview document summarizing the current state of the 23 HEPA indicators across the EU as well as a detailed factsheet for each Member State. Data collected in 2018 were also summarized in the form of four thematic factsheets: ‘Promoting PA in the health sector’,^17^ ‘Promoting PA in the education sector’,^18^ ‘Promoting physical activity in the sports sector’^19^ and ‘Promoting physical activity in the workplace’.^20^ The survey data have also been used for scientific publications that analysed overall trends in HEPA promotion in the EU across countries and across time,^21^^,^^22^ compared content and development processes of the national PA recommendations,^23^^,^^24^ and described monitoring of PA promotion in children and adolescents.^25^

### Evaluation of network outputs and benefits

**Figure 1:**
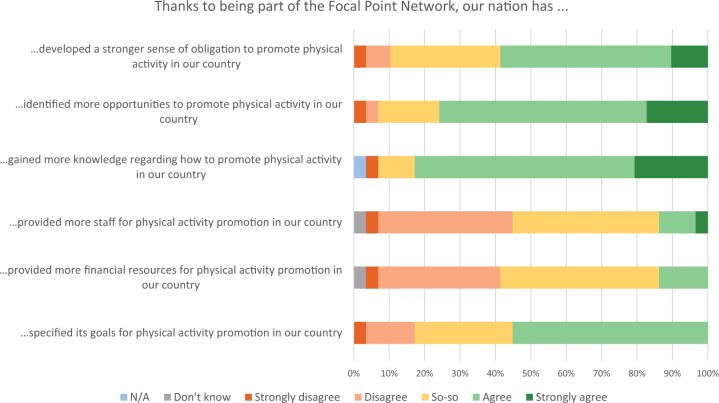
Focal points opinion on the effectiveness of participation in the Focal Point Network for national physical activity promotion

In total, 29 EU national focal points from 24 EU Member States filled out the online evaluation questionnaire. The main survey results are presented in table 3 and figure 1. The full text of the survey questions is provided in Supplementary appendix S1. The majority of focal points (82%) was generally satisfied with the network meetings.

**Table 3:** Main results of the evaluation survey among national focal points (*n* = 29 participants)

	Number	Percentage
Overall, how satisfied are you with the focal point meetings?		
Very satisfied	12	41
Satisfied	12	41
So-so	3	11
Not so satisfied	0	0
Not satisfied at all	0	0
No answer	2	7
How satisfied are you in general with the outputs of the Focal Point Network?		
Very satisfied	8	28
Satisfied	18	62
So-so	3	10
Not so satisfied	0	0
Not satisfied at all	0	0
Did you or your institution start any initiatives mainly because of the Focal Point Network?		
Yes	17	59
No	8	27
Do not know	4	14
Does the Focal Point Network contribute to people having more opportunities to be physically active in your country?		
Definitely	4	14
Highly likely	2	7
Likely	11	37.5
Unlikely	4	14
Highly unlikely	2	7
Definitely not	1	3.5
Do not know	5	17
Does the Focal Point Network contribute to people being more physically active in your country?		
Definitely	4	14
Highly likely	1	3.5
Likely	11	37.5
Unlikely	4	14
Highly unlikely	2	7
Definitely not	1	3.5
Do not know	6	20.5
Should nations in other world regions or other policy areas set up similar Focal Point Networks?		
Yes	27	93
No	0	0
Not sure	2	7

All focal points were at least partially satisfied with outputs of the Focal Points Network (3 answered ‘so-so’, 18 ‘satisfied’ and 8 ‘very satisfied’). The majority (72%) named the PA Country Factsheets as the most helpful output. According to the respondents, the factsheets support the process of gathering information about HEPA promotion at country level, give helpful insights on developments across the EU, and allow for a comparison of countries within the region and the exchange of experience. Focal points also used them in their communication with national policymakers, for the exchange with other sectors, and as a basis for policymaking. Some focal points mentioned that the most helpful output for their work were the connections inside the network and the opportunity to share their experience with colleagues during meetings and group discussions.

A majority (59%) of the focal points stated that their institutions started certain initiatives primarily as a result of the Focal Points Network’s activities. Focal points mentioned that participating in the network helped their institutions to start collaboration projects with other national organizations, sparked the development of working groups to solve specific PA promotion issues (e.g. sport in schools, PA opportunities for elderly, PA a work place) and (in four countries) also contributed to starting a development process for national PA recommendations. When asked about the main effects of their participation, they mentioned that it helped them specify goals for PA promotion, gain more knowledge about how to promote PA, identify more opportunities for PA promotion in their country and join or initiate collaborative projects with other countries.

More than half of the focal points felt that the network contributed to people having more opportunities for PA and being more physically active in their countries. Despite this positive overall assessment of the network, focal points saw several issues that require improvement. Some participants did not note any significant changes in HEPA promotion in their countries due to their participation in the network. Likewise, results imply that almost a third of focal points did not identify any new initiatives in their organization related to HEPA promotion following the establishment of the network. Also, the network does not seem to have had a significant impact on channelling additional funding or human resources into HEPA promotion.

Twenty-seven out of 29 focal points agreed that they would recommend setting up similar networks in other world regions or other policy areas. They perceived the sharing of experience and best practices between countries as beneficial, especially where countries have similar problems in PA promotion. The two interviewed experts from WHO and the EC also highlighted the effectiveness of the network. In the opinion of the WHO expert, it is a prime example of successful cooperation, as it both allows WHO to work closely with Member States and is beneficial for countries for gathering expertise in HEPA promotion. For the expert from the EC, a main benefit of the network is that it imposes certain monitoring obligations on Member States and also is used as a platform for exchange of expertise and best practice in PA promotion. The experts noted that the informal environment that has developed within the network is an advantage and allows for finding common solutions to problems in the field of PA promotion in the region, as well as for the exchange of successful practices.

### Possible improvements and extension of the network

Regarding ways to improve the efficiency of the Focal Points Network, focal points mentioned in the evaluation survey that they would like to have more opportunities to exchange experiences. They suggested using regular online conferences and web-based platforms as a potential solution. Three respondents pointed out that they would like to receive more support for collaboration with other sectors, such as organizing joint meetings of the health and sport sectors or involving the social sector to increase public awareness about the benefits of PA.

The experts from WHO and the EC agreed that a next development step for the network could be its extension to additional countries in the WHO European Region. The representative of the EC also highlighted that a more precise formulation of goals and deadlines for their implementation could help further improve the effectiveness of the network.

## Discussion

This article has attempted to provide an overview of how the EU Physical Activity Focal Points Network was set up, how it operates, what outputs it has produced and how its members perceive its benefits. The analysis shows that the network may serve as an example of successful cross-country collaboration in PA promotion, while results of the network’s performance assessment also point towards potential ways for further improvement.

The network has been able to contribute to policy monitoring, in particular with respect to the implementation of the EU Recommendation on HEPA, but also regarding PA promotion in the EU in general. Following the appointment of the focal points in 2014, three rounds of the survey on the 23 indicators of the HEPA Monitoring Framework were successfully conducted and published in the form of PA Country Factsheets and/or scientific articles.

According to the survey participants, the network also had positive effects on national PA promotion efforts and on cooperation between countries. In this context, it is worth noting that the network was originally created to perform one main function—the implementation of the HEPA Monitoring Framework—but that, over time, it has become a much more general platform for the exchange of experiences between the focal points from all EU countries.

An important factor for the success of the Focal Points Network appears to be the support of international organizations, i.e. the EC and WHO. As mentioned by the two experts interviewed, the establishment of the network was the result of a long collaboration that gave the two organizations important experience in conducting joint projects and distributing tasks for setting up and operating the network: the EC was more responsible for meeting organization and WHO more for providing expert consultations and conducting the surveys on the 23 indicators of the HEPA Monitoring Framework.

In contrast to the HEPA Europe network, which is also coordinated by WHO but focuses on scientific cooperation on a voluntary basis,^26^ EU Member States have made some commitments when forming the Focal Points Network, esp. nominating a contact officer within a given time frame and participating in HEPA indicator monitoring on a regular basis. It is also noteworthy that, by taking part in the network, Member States have given their implicit consent to being compared to other countries regarding their PA promotion and policy performance, as the Monitoring Framework allows for tracking progress on HEPA indicators across the entire EU in a comparable fashion.

### Limitations

We acknowledge that this study has some limitations. The results of the evaluation survey have shown that members highly appreciate the impact of the network on PA promotion policy at both the European and national level. However, it should be noted that these results are based exclusively on subjective self-reports by the focal points and the representatives of the EC and WHO, and that they thus do not allow for drawing direct conclusions about the actual impact of the network’s activities on PA promotion or even population-level PA behaviour in the EU. Consequently, it might be useful to add more objective methods to future assessments of the network’s performance and the progress of PA promotion in Europe, although this may involve both methodological and resourcing challenges.

### Future directions

The experiences of the EU PA Focal Points Network provide an opportunity to reflect upon two issues. First, could this network be a model for other world regions? And second, how could the effectiveness of such a network be increased over time?

Regarding the first question, one has to acknowledge that the EU seems to be exceptionally well-equipped to establish such a network. EU nations are politically integrated to a much higher degree than any other comparable set of countries in the world, they have a long history of collaborating on policymaking, there are existing regulatory frameworks that enable the EU to promote this type of collaboration,^27^ and funds are available from the EC to sustain networks for extended periods of time. It might be more difficult to set up comparable networks in other world regions where none or only some of these prerequisites are fulfilled. Nevertheless, this might still be feasible provided international organizations such as WHO invest sufficient political and technical resources to support such endeavours, and the WHO Regional Office for Europe has taken steps to extend the network to include additional countries in the Region from outside the EU.

The second question immediately raises the issue of the effectiveness of such networks and of how their impact might be increased. Besides the mandatory tri-annual surveys, the Focal Points Network is based largely on voluntary exchange at its semi-annual meetings. Thus, the network provides State Members with an opportunity to engage or disengage much to their own liking. In the future, this might lead to calls for a deeper integration of the network’s PA promotion efforts using more mandatory elements, e.g. a harmonization of national policies for PA promotion or larger financial contributions by the EC to sustain the network and stimulate national policy development. At this point, such a development seems rather unlikely as it requires changes to the mission statements of the HEPA focal points, but potential evidence for the effectiveness of such networks in stimulating policy development plus increasing (cost) pressures to combat sedentary lifestyles could open up such discussions in the future.

## Supplementary data

Supplementary data are available at *EURPUB* online.

## Funding

This research was conducted as part of a project funded by the German Federal Ministry of Health (ZMVI1-2520WHO001 and ZMVI1-2521WHO001). The ministry was neither involved in writing this manuscript nor in the decision to submit the article for publication.

## Disclaimer

The writing group takes sole responsibility for the content of this article, and the content of this article reflects the views of the authors only. K.W. and S.W. are staff members of the WHO. The authors affiliated with the World Health Organization (WHO) are alone responsible for the views expressed in this publication and they do not necessarily represent the decisions or policies of the WHO.

*Conflicts* *of* *interest*: None declared.

Key points

- The EU PA Focal Points Network has facilitated three rounds (2015, 2018 and 2021) of national data collection for the EU’s HEPA Monitoring Framework.
- The main outputs of the network are the PA Country Factsheets, connections created between network members and the opportunity to share experience with colleagues during meetings and group discussions.
- The findings of this study show that the PA Focal Points Network may serve as an example of successful cross-country collaboration in PA promotion, while the network’s performance assessment points towards potential ways for further improvement.
- A closer investigation of the networks’ effectiveness might be useful. This could also include work to support the network in formulating goals and implementation processes to reach these goals.

## Supplementary Material

ckac079_Supplementary_Data
