# Supplementary material for: Cross-country collaboration for physical activity promotion: experiences from the European Union Physical Activity Focal Points Network
Source: Eur J Public Health. 2022 Aug 26;32(Suppl 1):i14–21. doi: 10.1093/eurpub/ckac079 (PMC9421415; doi:10.1093/eurpub/ckac079)
Supplement: ckac079_Supplementary_Data [file ckac079_supplementary_data.docx]

**Appendix 1.** Questionnaire on satisfaction with/effectiveness of the network

| **Question** | **Answer options/comments** |
| --- | --- |
| *1 Thank you for taking part in our survey on the EU/WHO Physical Activity Focal Point Network! We would like to start by asking you to provide some information about yourself and the network meetings you attended.* |  |
| Q_1a What is your name? | Open |
| Q_1b Which country do you represent? | Open/drop down |
| Q_1c Which organization do you work for? | Open |
| Q_1d What is your position within this organization (job title, description)? | Open |
| Q_1e Which of the meetings of the EU PA FP Network did you attend personally? | List of all meetings |
| Q_1f For which of the meetings did another representative of your organization or government stand in for you? | List of all meetings |
| *2 Next, we would like to know how you perceived the meetings of the Focal Point Network.* |  |
| Q_2a Overall, how satisfied are you with the Focal Point Meetings? | Very satisfied, satisfied, so-so, not so satisfied, not satisfied at all |
| Q_2b How satisfied are you with the preparation of the meetings by EU/WHO? | Very satisfied, satisfied, so-so, not so satisfied, not satisfied at all |
| Q_2c How satisfied are you with the composition of the group and the perspectives represented at the meetings? | Very satisfied, satisfied, so-so, not so satisfied, not satisfied at all |
| Q_2d How satisfied are you with the agenda, the topics covered, the opportunities for exchange and networking at those meetings? | Very satisfied, satisfied, so-so, not so satisfied, not satisfied at all |
| Q_2e If you look back, which FP meeting has in your opinion been the most productive? | List of all meetings |
| Q_2f Please briefly explain your choice. | Open |
| *3 The Focal Point Network has produced a number of outputs such as mailing lists, online discussions, country factsheets beside the meetings. We would like to know your opinion on these outputs.* |  |
| Q_3a How satisfied are you in general with the outputs of the Focal Point Network? | Very satisfied, satisfied, so-so, not so satisfied, not satisfied at all |
| Q_3b In your opinion, which output has been most helpful for your work? | Open |
| Q_3c Please briefly explain your choice. | Open |
| *4 We would now like to get your opinion about the effects of the network on your work and public policy in your country.* |  |
| Q_4a Did you or your organization start any initiatives mainly because of the Focal Point Network? Please think about things such as organizing workshops or meetings, producing briefings for policymakers, or supporting the development of legislation. | Yes/no  If no, continue to Q_4c |
| Q_4b Please name these initiatives, briefly describe them, and provide links/references where available. | Open |
| Q_4c Please give us your opinion on the following statements: Since becoming part of the Focal Point Network, my organization has … |  |
| Q_4c_1 …specified its goals for physical activity promotion in our country | Strongly agree, agree, so-so, disagree, strongly disagree |
| Q_4c_2 …provided more financial resources for physical activity promotion in our country | Strongly agree, agree, so-so, disagree, strongly disagree |
| Q_4c_3 …provided more staff for physical activity promotion in our country | Strongly agree, agree, so-so, disagree, strongly disagree |
| Q_4c_4 …gained more knowledge regarding how to promote physical activity in our country | Strongly agree, agree, so-so, disagree, strongly disagree |
| Q_4c_5 …identified more opportunities to promote physical activity in our country | Strongly agree, agree, so-so, disagree, strongly disagree |
| Q_4c_6 …developed a stronger sense of obligation to promote physical activity in our country | Strongly agree, agree, so-so, disagree, strongly disagree |
| Q4_d Thinking beyond your own organization and about the general effects that the Focal Point Network might have had on physical activity promotion in your country, please give us your opinion on the following statements: Thanks to being part of the Focal Point Network, our nation has… |  |
| Q_4d_1 …specified its goals for physical activity promotion in our country | Strongly agree, agree, so-so, disagree, strongly disagree |
| Q_4d_2 …provided more financial resources for physical activity promotion in our country | Strongly agree, agree, so-so, disagree, strongly disagree |
| Q_4d_3 …provided more staff for physical activity promotion in our country | Strongly agree, agree, so-so, disagree, strongly disagree |
| Q_4d_4 …gained more knowledge regarding how to promote physical activity in our country | Strongly agree, agree, so-so, disagree, strongly disagree |
| Q_4d_5 …identified more opportunities to promote physical activity in our country | Strongly agree, agree, so-so, disagree, strongly disagree |
| Q_4d_6 …developed a stronger sense of obligation to promote physical activity in our country | Strongly agree, agree, so-so, disagree, strongly disagree |
| Q_4e Have there been any other effects of the Focal Point Network on your work as a Focal Point or activities in your country in general that you would like to point out? Please briefly name and describe them. | Open |
| *5 Next, we would like to get your opinion about the effects of the network on the population’s physical activity in your country.* |  |
| Q_5a Does the focal point network contribute to people having more opportunities to be physically active in your country? | Definitely, highly likely, likely, unlikely, highly unlikely, definitely not |
| Q_5b Although it might be difficult to answer, to the best of your knowledge, does the Focal Point Network contribute to people being more physically active in your country? | Definitely, highly likely, likely, unlikely, highly unlikely, definitely not |
| *6 Taking part in the Focal Point Network and its meetings implies an investment of time and other resources. We would now like to have your opinion on how you perceive the relation of costs and benefits of the network.* |  |
| Q_6a All in all, how would you rate the effectiveness of your country in being part of the FP network. | The benefits greatly outweigh the costs;  the benefits outweigh the costs;  the benefits slightly outweigh the costs;  the benefits and costs are about equal;  the costs slightly outweigh the benefits;  the costs outweigh the benefits;  the costs greatly outweigh the benefits |
| Q_6b In your opinion, what could be done to improve the efficiency of the Focal Point Network? | Open |
| *7 Finally, we would like to get your opinion on whether you believe the Focal Point Network could serve as an inspiration for other groups of countries or policy areas.* |  |
| Q_7a In your opinion, should nations in other world regions or other policy areas set up similar focal point networks? | Yes, no, not sure |
| Q_7b If other nations or policy areas would set up their own focal point networks, what advice would you have for them? | Open |

Note: In the survey, respondents were asked to indicate their name, organization and position (Q1a, Q1b, Q1c). This was done for the following reasons:

(a) The information was important to verify who was actually present at individual Focal Points Network Meetings.

(b) It allowed us to sort data by country.

(c) It helped identify the different sectors involved in the network.

(d) Making only part of the survey fully anonymous would have been very difficult with the limited resources available for this study.

While this procedure may have impacted respondents’ answers to the subsequent questions (esp. sections 2 and 3), respondents were not obliged to reveal their personal information, and the final results were published in a way that does not enable readers to trace back answers to specific individuals
